# A Novel *SLC27A4* Splice Acceptor Site Mutation in Great Danes with Ichthyosis

**DOI:** 10.1371/journal.pone.0141514

**Published:** 2015-10-27

**Authors:** Julia Metzger, Anne Wöhlke, Reinhard Mischke, Annalena Hoffmann, Marion Hewicker-Trautwein, Eva-Maria Küch, Hassan Y. Naim, Ottmar Distl

**Affiliations:** 1 Institute for Animal Breeding and Genetics, University of Veterinary Medicine Hannover, Hannover, Germany; 2 Clinic for Small Animals, University of Veterinary Medicine Hannover, Hannover, Germany; 3 Department of Pathology, University of Veterinary Medicine Hannover, Hannover, Germany; 4 Department of Physiological Chemistry, University of Veterinary Medicine Hannover, Hannover, Germany; Ohio State University Medical Center, UNITED STATES

## Abstract

Ichthyoses are a group of various different types of hereditary disorders affecting skin cornification. They are characterized by hyperkeratoses of different severity levels and are associated with a dry and scaling skin. Genome-wide association analysis of nine affected and 13 unaffected Great Danes revealed a genome-wide significant peak on chromosome 9 at 57–58 Mb in the region of *SLC27A4*. Sequence analysis of genomic DNA of *SLC27A4* revealed the non-synonymous SNV SLC27A4:g.8684G>A in perfect association with ichthyosis-affection in Great Danes. The mutant transcript of *SLC27A4* showed an in-frame loss of 54 base pairs in exon 8 probably induced by a new splice acceptor site motif created by the mutated A- allele of the SNV. Genotyping 413 controls from 35 different breeds of dogs and seven wolves revealed that this mutation could not be found in other populations except in Great Danes. Affected dogs revealed high amounts of mutant transcript but only low levels of the wild type transcript. Targeted analyses of SLC27A4 protein from skin tissues of three affected and two unaffected Great Danes indicated a markedly reduced or not detectable wild type and truncated protein levels in affected dogs but a high expression of wild type SLC27A4 protein in unaffected controls. Our data provide evidence of a new splice acceptor site creating SNV that results in a reduction or loss of intact SLC27A4 protein and probably explains the severe skin phenotype in Great Danes. Genetic testing will allow selective breeding to prevent ichthyosis-affected puppies in the future.

## Introduction

Hereditary ichthyoses represent a heterogenous group of skin cornification disorders that affect the entire integument and are characterized by hyperkeratosis and blistering or scaling [1, 2]. In human, a wide variety of mutations have been shown to be responsible for different types of ichthyoses [3, 4]. They were classified into syndromic and non-syndromic forms and further subgroups with regard to clinical signs, onset of the disease and genetic background. On the whole 36 genes have been detected which were supposed to be involved in non-syndromic and/or syndromic human ichthyoses [1]. For the investigation of the causative mutations for human ichthyoses domestic animals have been shown to be useful model organisms. Ichthyosiform dermatoses have been described in cattle, mice, rats, dogs and pigs [5–15].

In dogs, several ichthyoses-associated mutations have been detected in specific breeds which revealed in most cases an autosomal recessive mode of inheritance [10–13, 15]. In Golden Retrievers lamellar ichthyosis was shown to be associated with a mutant *PNPLA1 (patatin-like phospholipase domain containing 1)* which was supposed to harbor potential causative mutations for human ichthyosis in European and North African populations as well [4, 10]. A similar type of lamellar ichthyosis with a generalized severe hyperkeratosis was detected in Jack Russell Terriers. A 1980-bp insertion in *transglutaminase 1 (TGM1)* could be shown to disrupt splicing of exon 10 and result in a premature stop codon and a significant decrease of mRNA expression [13, 16]. In Great Danes, a primary disorder of cornification has been detected in different litters as well. Patho-histological examinations indicated a congenital non-epidermolytic ichthyosis of a lamellar type [15]. A close relationship in-between affected dogs in pedigree analysis suggested an ichthyosis type resembling the autosomal recessive ichthyosis in humans.

The objective of this study was to perform a genome-wide association analysis and candidate gene study in order to elucidate the genetic background of ichthyosis in Great Danes and to identify the potential causative mutation.

## Results

### Phenotype

We examined 15 cases of Great Dane puppies affected with ichthyosiform dermatosis from five different litters. All affected dogs were Fédération Cynologique Internationale (FCI) breed standard Great Danes of the color type black/harlequin and were all distantly related (S1 Fig). The familial pattern of the 15 cases supports a recessive simple Mendelian mode of inheritance. Dominant and X-linked modes of inheritance can be excluded because parents were normal and both sexes were affected. All affected puppies shared one male ancestor.

**Fig 1:**
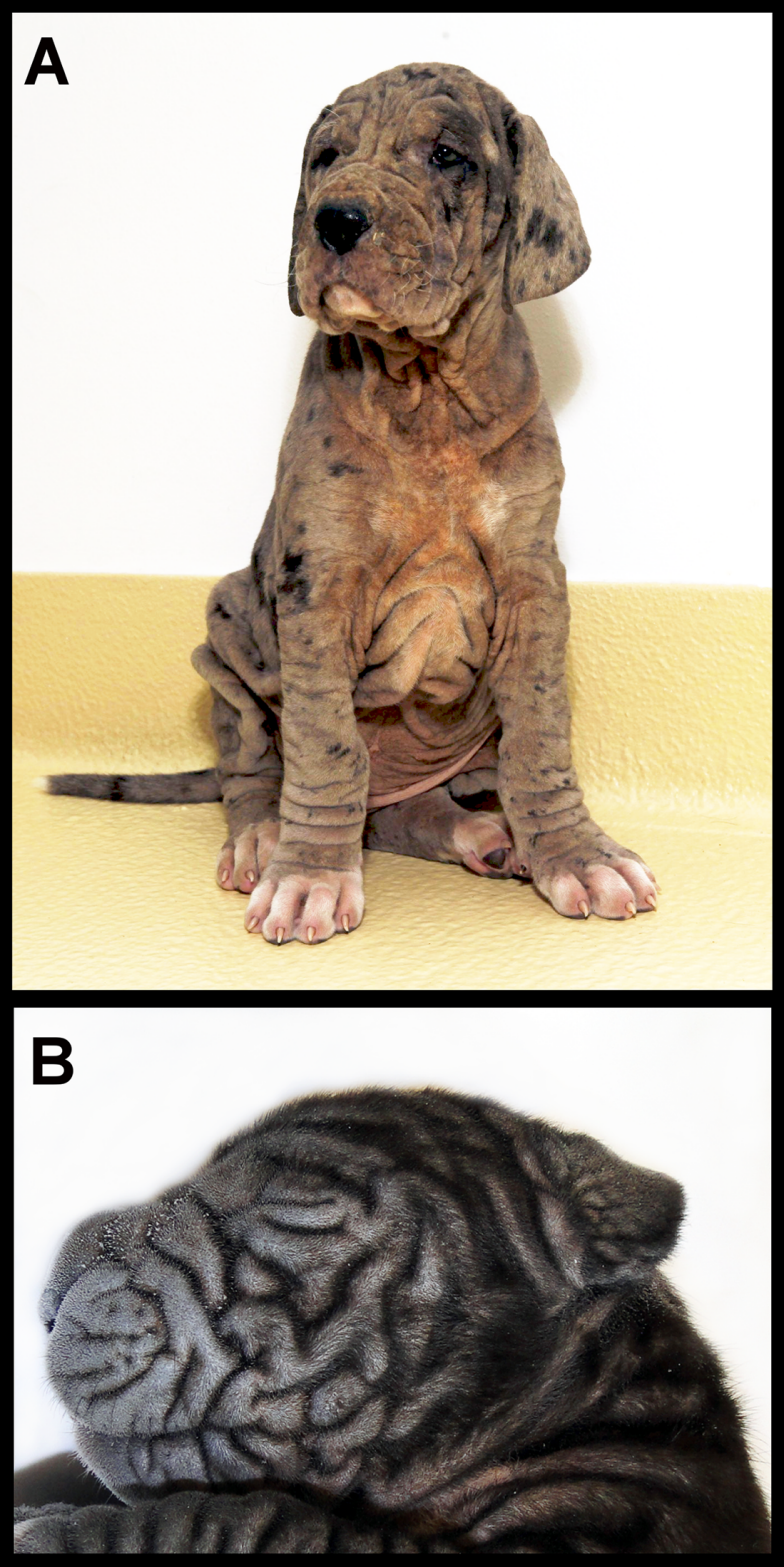
Clinical picture of ichthyosiform dermatosis in Great Danes. (A) A five week old puppy with a characteristically wrinkled and squamous skin is shown. (B) In a one week old puppy prominent wrinkles in the face impede the opening of the dog’s eyes.

Clinical examination revealed signs of a generalized severe hyperkeratosis in all cases with a formation of a strongly wrinkled, thickened and scaling skin especially in the region of the eyes and nose (Fig 1). These changes led to a dry inelastic and lichenified skin of an untidy appearance in the affected dogs and a markedly swollen periocular skin which impeded the opening of the puppy’s eyes in some cases. In-between the wrinkles the exudative character of the skin promoted secondary infections. Due to the poor prognosis, all affected dogs were euthanized at the age of 7–40 days. Additional computer tomographic and endoscopic examinations after euthanasia in two five week old affected dogs revealed a ventrally displaced auditory canal with an atypically wrinkled shape but no signs of other anomalies.

In addition to clinical examinations all affected dogs were carefully studied in necropsy [15]. The wrinkled skin showed a patho-histological feature of a marked epidermal and follicular hyperkeratosis (S2 Fig).

### Genome-wide association analysis

**Fig 2:**
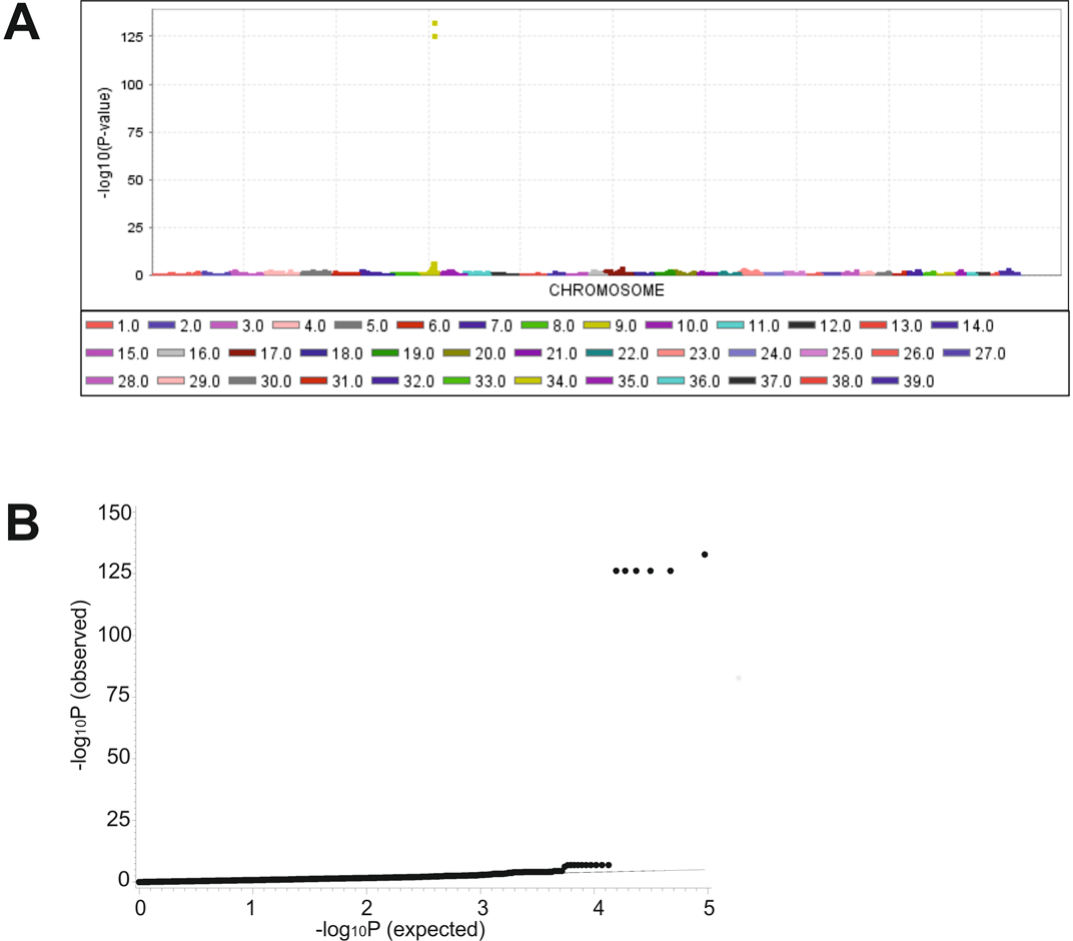
Genome-wide association analysis (GWAS) for ichthyosis in Great Danes. (A) Manhattan-plot of the -log_10_P-values from GWAS. A significant peak is located on canine chromosome (CFA) 9 in the region of 54,031,320–54,772,131 bp (CanFam3.1). (B) Q-Q plot of observed versus expected -log_10_P-values. Six SNPs show highly significant -log_10_P-values.

In order to map the locus underlying ichthyosiform dermatosis, a genome-wide association study (GWAS) was performed using 93,882 SNPs in nine affected and 13 unaffected Great Danes. A genome-wide significant association was detected on canine chromosome (CFA) 9 for a region of 740 kb from 54,031,320 to 54,772,131 bp on the dog genome assembly CanFam3.1 (Fig 2). The highest P-values reached six SNPs (P-value = 1e-126.3 to 1e-132.8) located within this region. The quantile-quantile (Q-Q) plots illustrated that inflation due to stratification effects had not increased P-values. After correction for cryptic relatedness, an association for the same genomic region was confirmed showing the highest peaks for these SNPs (P-values from 1e-88.1 to 1e-94.3). Removing of these most significant SNPs did not reveal a further genome-wide significantly associated region. All six associated SNPs within this genomic region showed a perfect co-segregation with the phenotype (S1 Table). The nine affected dogs were homozygous and the unaffected dogs were heterozygous (n = 10) or homozygous for the alternate allele (n = 3). All obligate carriers (parents of affected dogs, n = 3) were heterozygous. The minor allele frequency (MAF) of these highly associated SNPs was 0.38 for all Great Danes, 0.0 for affected and 0.36 for unaffected dogs. We used a candidate gene approach and investigated all 16 genes in the associated region and further 37 genes at a distance of 1 Mb from this associated region for known involvement in ichthyosis affection in mammals. Adjacent to this significantly associated region, *SLC27A4 (Solute Carrier Family 27)* is annotated at 55,164,817–55,177,741 bp (Ensembl, http://www.ensembl.org), approximately 393 kb downstream of this region.

We analyzed the haplotype structure and haplotype association of this associated genomic region. The six highly associated SNPs are located within two different haplotype blocks (S3 Fig). Two SNPs (BICF2S23340470, BICF2G630473300) are located within a haplotype block at 54,010,938–54,144,992 bp of 134 kb, whereas the other four highly associated SNPs (BICF2G630473617, BICF2G630473695, BICF2G630473702, BICF2G630473744) can be found within a haplotype block at 54,426,765–54,772,131 bp of 343 kb length. Haplotypes with the disease-related alleles were significantly associated (P-value <0.0001) with the affection status.

### Sequence and mutation analysis

**Fig 3:**
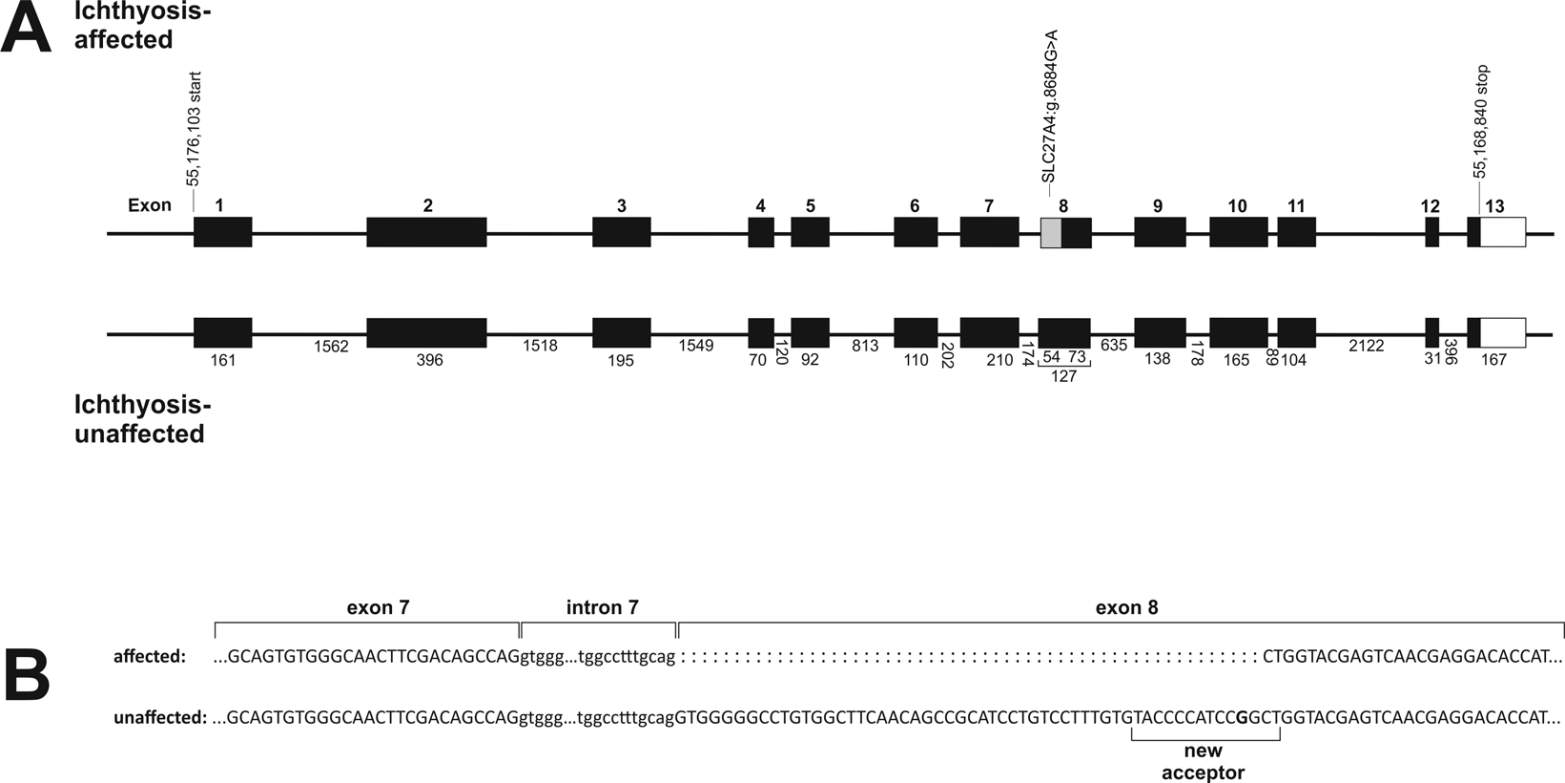
Experimental data derived from Sanger sequencing of *SLC27A4* complementary DNA. (A) Gene model of ichthyosis-affected in comparison to unaffected Great Danes. In the region of the non-synonymous SNV SLC27A4:g.8684G>A affected dogs show aberrantly spliced transcript with an in-frame loss of the first 54 bp of exon 8. (B) A new acceptor site is created by the A-allele of SLC27A4:g.8684G>A that results in a shorter RNA product. Affected dogs show only a small amount of RNA of the wild type size.

Sequence analysis of exons and exon/intron boundaries of *SLC27A4* revealed two variants, the exonic SNV *SLC27A4*:g.8684G>A (ss1536958154) and the intronic indel *SLC27A4*:g.9852del (ss1536958155, Table 1). Functional analysis of the indel showed no significant reference to any modifying effect like a variation of acceptor sites that could be induced by this variant. However, the SNP *SLC27A4*:g.8684G>A in exon 8 was predicted to be a non-synonymous mutation resulting in a substitution of glutamine for arginine (p.Arg417Gln). The incorporation of a polar and neutral amino acid instead of a basic amino acid in this region was predicted to be deleterious with a probability of approximately 50% (subPSEC score = -2.5, substitution position-specific evolutionary conservation). Human splicing finder [17] tool suggested that this mutation within exon 8 created a new splice acceptor site motif ‘taccccatccagCT’, located 52 bp downstream of the authentic 3’ splice acceptor site (Fig 3).

**Table 1:** Variants detected by sequencing exons and intron boundaries of the genomic and complementary DNA (cDNA) of *SLC27A4* in Great Danes.

SNV name	Type	Base change	Predicted effect	Amino acid change
SLC27A4:g.8684G>A	SNV	G>A	non-synonymous	R>Q
SLC27A4:g.9852delC	Deletion	C	intron	N/A

**Fig 4:**
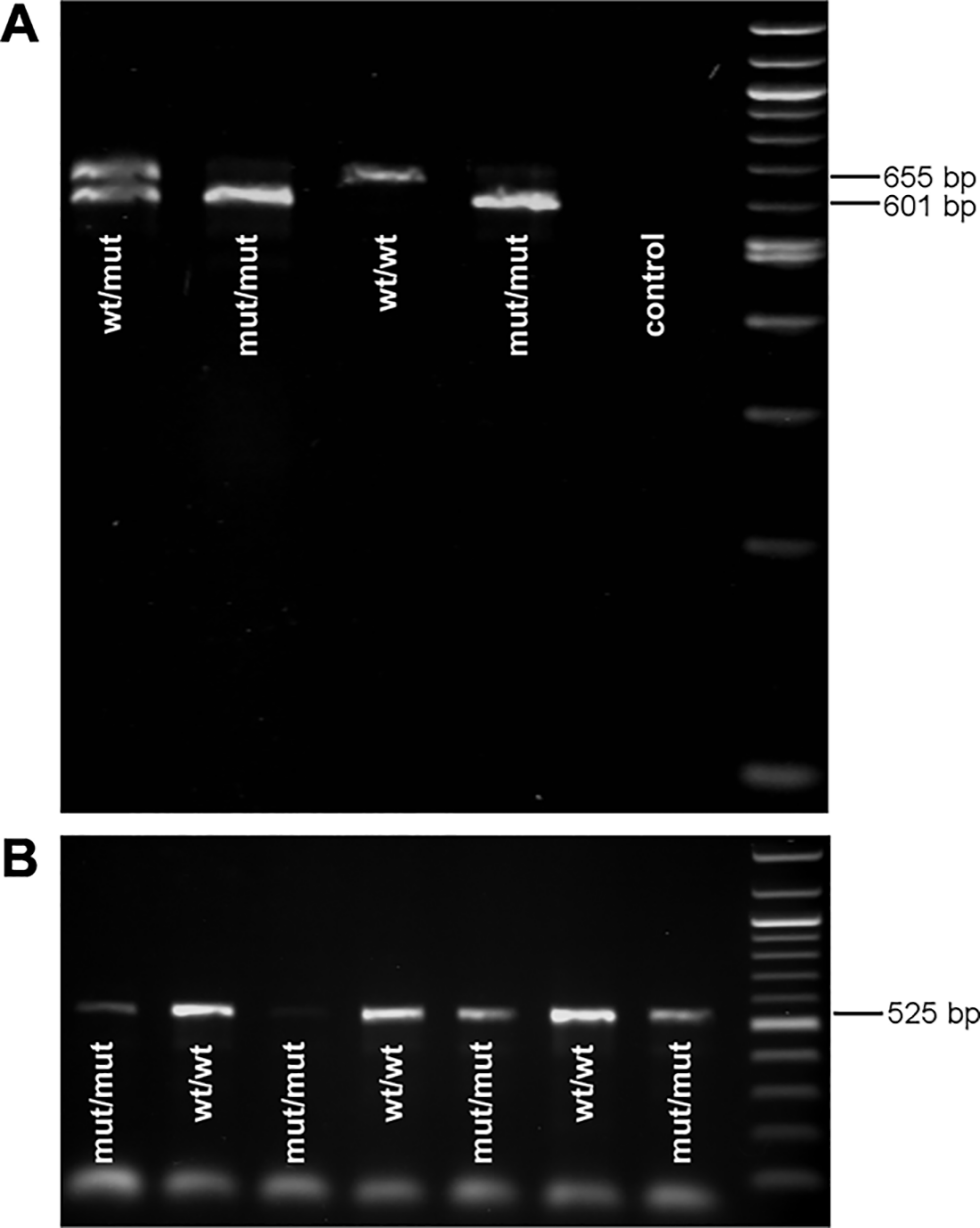
Mutation analysis of *SLC27A4* using complementary DNA. (A) A loss of 54 bp is present in affected Great Danes homozygous for allele A (mut/mut) in the SNV *SLC27A4*:g.8684G>A. The PCR-product spans 601 bp from exon 7 to 11 in affected dogs and 655 bp in unaffected dogs homozygous for the wild type (wt/wt) whereas heterozygous dogs show both products (wt/mut). A very small amount of wild type PCR-product (655 bp) can also be seen in affected dogs. (B) PCR-product of affected and unaffected Great Danes using a primer placed into the first 54 bp of exon 8. It confirms that affected dogs also have small amount of RNA of wild type size.

Sequencing of the complementary DNA (cDNA) of *SLC27A4* revealed a loss of 54 bp from the beginning of exon 8 in affected dogs, and thus an exon 8 of 73 bp in length, whereas unaffected dogs showed an exon 8 of 127 bp in length as expected by the reference sequence. The differences in the size of exon 8 in-between affected and unaffected dogs could be significantly shown by PCR-products spanning the affected region (Fig 4). Obligate carriers revealed two different PCR-products. Nevertheless, some affected dogs also showed a very weak band of the wild type size. Placing an additional cDNA-primer directly into the 54 bp from the 5’ end of exon 8 confirmed that also low amounts of wild type transcript could be amplified in all affected dogs. Sequencing of this wild type PCR-product in affected dogs confirmed that it was identical with the wild type transcript in unaffected dogs. The experimental sequence showed an identity of 98.55% with Ensembl (ENSCAFT00000031925, transcript 2) and 100% with the NCBI (XM_548438.4) reference sequence. The predicted transcript 1 sequence of *SLC27A4* provided from Ensembl (ENSCAFT00000049296) could not be amplified in any of the investigated hair root samples of affected and unaffected dogs. Nevertheless, NetStart 1.0 (http://www.cbs.dtu.dk/services/NetStart/) suggested a shorter open reading frame of *SLC27A4* as expected by the reference sequence starting at position 169 (NCBI XM_548438.4).

### Protein expression

**Fig 5:**
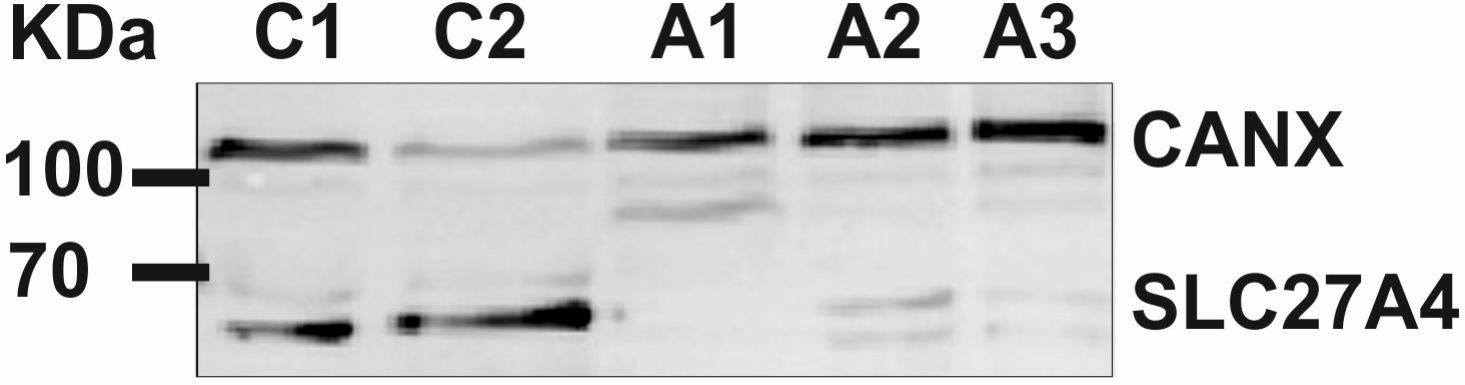
Western blot analysis of SLC27A4 in affected and unaffected Great Danes. Wild type SLC27A4 was detected at 66.09 kDa and mutant SLC27A4 at 64.11 kDa using a polyclonal anti-SLC27A4 antibody corresponding to a region within amino acids 1 and 202 of the dog SLC27A4. Anti-Calnexin (CANX) polyclonal antibody was used as control. Affected Great Danes (A1-A3) show either low or no protein levels of mutant and wild type SLC27A4. In unaffected controls (C1-C2) only wild type protein is expressed.

Protein analysis was performed to estimate the consequences of the 54 bp deletion and thus a loss of 18 amino acids (p.Val344_Arg361del) on SLC27A4 protein expression. Samples of skin tissue protein from affected dogs revealed either low or no SLC27A4 protein levels of expected wild type size (66.09 kDa) and very low levels of mutant protein (64.11 kDa) in western blot analysis. In unaffected dogs only wild type SLC27A4 protein could be shown to be expressed matching the experimental protein size of 66.09 kDa (Fig 5). Functional analysis of SLC27A4 using the protein sequence and classification tool InterPro suggested that the truncation leads to a size reduction of the AMP-dependent synthetase/ligase domain (IPR000873) [18, 19].

### Validation

Both mutations detected in *SLC27A4* were genotyped in 28 Great Danes including the detection sample (n = 22) and further six Great Danes with patho-histologically confirmed ichthyosis. We found a perfect association of the SNV *SLC27A4*:g.8684G>A with the affected phenotype but no segregation of the phenotypes for the indel *SLC27A4*:g.9852del. Additional verification of *SLC27A4*:g.8684G>A in further 220 Great Danes, 413 controls of 35 different dog breeds and seven wolves confirmed the perfect association (Table 2) and a total absence of the disease-associated allele A in any other tested dog breed or wolves (S2 Table).

**Table 2:** Distribution of the *SLC27A4*:g.8684G>A genotypes in 249 Great Danes, 413 controls of other breeds, and seven wolves.

Animals	G/G	G/A	A/A
Ichthyosis-affected (n = 15)	0	0	15
Obligate carriers (n = 5)	0	5	0
Unaffected relatives (n = 85)	47	38	0
Controls—Great Danes (n = 144)	144	0	0
Controls—other breeds (n = 413)	413	0	0
Controls—wolves (n = 7)	7	0	0

## Discussion

In this study a genome-wide association analysis for autosomal recessive ichthyosis in Great Danes showed a highly significant peak on chromosome 9 near *SLC27A4*. Sequencing of the genomic DNA of *SLC27A4* revealed the exonic variant *SLC27A4*:g.8684G>A, 52 bp downstream of the beginning of exon 8, which was proposed to create a new and strong splice acceptor site, and thus an in-frame loss of 54 bp in the *SLC27A4* transcript of ichthyosis-affected puppies. We found that all 15 ichthyosis-affected Great Danes were homozygous for *SLC27A4*:g.8684G>A mutant allele A whereas none of the genotyped 413 dogs of 35 different breeds and seven wolves showed a SNV allele different from the wild type. Affected dogs could be shown to express low or no levels of mutant truncated protein and very low or no levels of wild type SLC27A4 protein as well.

Functional analyses of SLC27A4 revealed this protein to be the major enzyme for activating very long chain fatty acid substrates [20]. SLC27A4 was shown to drive indirectly the uptake of fatty acids at the plasma membrane by catalyzing esterification from its location in the endoplasmatic reticulum [21]. Analyses of skin biopsies in ichthyosis patients suggested the mutant SLC27A4 protein to induce an uneven distribution of lipids and disturb the formation of the skin barrier [22]. The importance of *SLC27A4* for skin and hair development was also demonstrated by the mouse model of wrinkle-free mice that revealed a defective skin barrier by a retrotransposon insertion in *SLC27A4* [23]. Fatp4^-/-^ mice showed a thick and extremely tight skin similar to the human restrictive dermopathy [23, 24]. In our study, we could show that a splice site modifying mutation and a subsequent lack or marked reduction of SLC27A4 wild type protein could also play a crucial role for skin developmental defects in dogs.

Modifying splice site mutations affecting SLC27A4 protein could also be observed in human. Human *SLC27A4*, the *fatty acid transport protein 4 (FATP4)*, was shown to play a role in ichthyosis prematury syndrome [22, 25]. Sequence analyses of affected patients from North Africa, Middle East and Scandinavia revealed in total ten mutations in *SLC27A4* that could not be found in healthy controls [22, 25]. Two of these mutations were predicted to result in splice site changes which in turn induce the truncation of SLC27A4 protein. Patients homozygous for p.C168X were even supposed to express no SLC27A4 protein showing no detectable band of SLC27A4 in western blot analysis [22]. Nevertheless, none of the mutations found in human were located in the homologous region near SLC27A4:g.8684G>A detected in Great Danes.

Our results of western blot analysis suggest that due to low transcription of wild type RNA in affected dogs, only a small amount or no stable intact SLC27A4 protein was produced. However, a high amount of mutant transcript with an in-frame loss of 54 bp could be verified in skin tissues in affected dogs. We propose that the strong decrease of protein levels was a result of degradation of the major proportion of the resulting shortened protein by the ubiquitin-proteasome pathway. Analyses in human gynecological cancer cells suggested in-frame insertions or deletions to have a strong impact on the stability of proteins [26]. Normal mRNA levels of *AT-rich interactive domain 1A* resulted in decreased protein levels due to increased proteasomal degradation. Quality-control mechanisms have been shown to be sensitive systems in eukaryotic cells which have been able to ensure quality of intracellular proteins and to degrade mutant proteins that could considerably disturb folding processes [27]. Defects in this sensitive system were proposed to result in various malfunctions in skin regulative processes. We suppose that the impact of the truncation in the AMP-dependent synthetase/ligase domain of *SLC27A4* was markedly high and could have negatively influenced protein function. In human Dorfman-Chanarin syndrome, a truncation of CGI-58 protein could be shown to affect the lipase/esterase/thioesterase activity of CGI-58 and result in severe ichthyosis [28]. A lack of protein expression could also be detected in Norfolk Terriers affected with ichthyosis [12]. It was supposed that the mutation in the consensus donor splice site of intron 5 resulted in a nonsense-mediated decay of *KRT10* transcripts. In lamellar ichthyosis-affected Jack Russell Terriers only a small amount of transglutaminase 1 (TGM1) protein could be detected on immunoblotting triggered by an insertion that disrupted the expression of *TGM1* [13]. It can be assumed that a reduced SLC27A4 protein expression could result in analogous skin damaging effects in Great Danes.

In conclusion, we identified a *SLC27A4* missense variant as the most likely causative mutation for ichthyosis in Great Danes. Together with data from Fatp4 transgenic mice our data confirm that a lack of SLC27A4 protein causes epidermal hyperplasia resulting from increased proliferation of suprabasal cells. Genetic testing of this mutation will open the opportunity for selective breeding to avoid mating among genetic carriers and producing ichthyosis-affected puppies.

## Materials and Methods

### Ethics statement

All animal work has been conducted according to the national and international guidelines for animal welfare. The Lower Saxony state veterinary office at the Niedersächsisches Landesamt für Verbraucherschutz und Lebensmittelsicherheit, Oldenburg, Germany, was the responsible Institutional Animal Care and Use Committee (IACUC) for this specific study. The EDTA-blood sampling of the Great Danes for the present study had been approved by the IACUC of Lower Saxony, the state veterinary office Niedersächsisches Landesamt für Verbraucherschutz und Lebensmittelsicherheit, Oldenburg, Germany (registration number 33.9-42502-05-14A465). Tissue samples were taken immediately after euthanasia of the affected puppies. EDTA-blood samples of control breeds derived from the bio-bank for diagnostic purposes and were taken from accredited veterinarians.

### Animals

For the GWAS, EDTA-blood samples of 22 Great Danes were employed. Validation of the mutations identified was performed in the detection sample (n = 22) and further six Great Danes with histo-pathologically confirmed ichthyosis. In addition, the SNV *SLC27A4*:g.8684G>A was validated in 221 EDTA-blood samples of unaffected Great Danes and 413 controls of 35 different breeds of dog and seven wolves (S2 Table). Skin tissues were collected from 6 affected Great Dane puppies that had to be euthanized due to the poor prognosis and two Great Dane controls euthanized due to other fatal diseases. All affected dogs were examined by veterinarians at the University of Veterinary Medicine Hannover. Euthanized animals were submitted for necropsy and histopathological examination to the University of Veterinary Medicine Hannover. Skin tissues from affected Great Danes and controls were fixed in 10% buffered formalin (pH 7.4) and embedded in paraffin. Sections of three μm were stained with hematoxylin and eosin (HE) [15].

### Genotyping

Genomic DNA was extracted using 500 μl EDTA-blood and a standard ethanol fraction [29]. Samples of nine dogs representing four litters with ichthyosis-affected puppies were available for analysis (S1 Fig). Genotyping of these 9 affected and 13 unaffected Great Danes was done using the canine Illumina high density beadchip (Illumina). Quality criteria for analyses of the 173,662 SNPs genotyped were minor allele frequencies (MAF) >0.05, genotyping rate per SNP >0.90 and tests for Hardy-Weinberg equilibrium (P-value<0.000001). After filtering for quality criteria, 93,882 SNPs have been left for analysis. The mean genotyping rate per individual was 99 percent.

Raw and processed data is available for the 22 Great Danes with genotypes and can be obtained through the GEO website (http://www.ncbi.nlm.nih.gov/geo/) using accession number GSE73400.

The variant S*LC27A4*:g.8684G>A identified within *SLC27A4* was genotyped in 249 Great Danes and 413 controls of 35 different dog breeds and seven wolves using restriction fragment length polymorphisms according to a standard PCR- and digestion-protocol (S3 Table) [30]. The indel mutation *SLC27A4*:g.9852del was visualized using gel electrophoresis on 6% polyacrylamide denaturing gels (RotiphoreseGel 40, Carl Roth, Karlsruhe, Germany) and an automated capillary sequencer (LI-COR 4300, LI-COR Biotechnology, Bad Homburg, Germany).

### Sequence analysis

The genomic regions of *SLC27A4* exons and exon/ intron boundaries as well as the cDNA of *SLC27A4* (473 bp 5’UTR to 477 bp 3’UTR) were sequenced in two ichthyosis-affected and two unaffected Great Danes. RNA was isolated from hair roots in stabilization RNALater reagent (Qiagen, Maryland, USA) and transcribed into cDNA according to standard protocols [30]. For complete DNA removal, RNA was additionally treated with DNase (RNase-Free DNase Set, Qiagen). Primers were designed using Primer3 (http://frodo.wi.mit.edu/primer3/) and PCR was prepared according to a standard mastermix protocol [30] using Q-solution (Qiagen) as enhancer reagent (S4 Table). The PCR-reaction for cDNA and genomic DNA was run on a thermocycler TProfessional 96 (Biometra, Göttingen, Germany) according to the following protocol: 95°C for 5 minutes, 38 cycles of 94°C for 30 seconds, primer specific annealing temperature for 30 seconds and 72°C for 45 seconds. Finally, the reaction remained for 5 minutes at 72°C and cooled down to 4°C. PCR-products were cleaned up using Exonuclease I and FastAP Thermosensitive Alkaline Phosphatase (Thermo Scientific, Darmstadt, Germany) according to the manufacturers’ protocol (http://www.thermoscientificbio.com/dna-and-rna-modifying-enzymes/exonuclease-i). Sequencing and alignments were performed by the automated sequencer Genetic Analyzer 3500 (Applied Biosystems by Life Technologies, Darmstadt, Germany) and the analysis software Sequencher 4.8 (Genes Codes, Ann Arbor, MI, USA).

### Western blot analysis

For western blot analysis we isolated protein from skin tissues of three affected and two unaffected Great Danes using Ultra Thurax Homogenizer. Equal protein amounts were loaded and separated by 12% SDS-PAGE and blotted onto a polyvinylidene fluoride membrane (Roti-PVDF, Carl Roth). A polyclonal rabbit anti-FATP4 antibody (Thermo Scientific) at a 1:1000 dilution was used for hybridization of the membrane. Anti-Calnexin antibody (CANX, 1:1000, Sigma-Aldrich Chemie GmbH, Munich, Germany) served as control for a second hybridization of the same membrane. After removing the unbound primary antibody, the membrane was exposed to anti-rabbit antibody at 1:5000 dilution (goat anti-rabbit IgG, Dianova, Hamburg, Germany) and detected by enhanced chemiluminescence (SuperSignal West Femto Chemiluminescent Substrate, Thermo Scientific) on a Gel Doc XR+ system.

### Bioinformatic analysis

Further estimation of the functional impact of the detected non-synonymous mutation on the protein was performed using Panther classification system (www.pantherdb.org) [31, 32] and Human Splicing Finder tool (version 2.4.1, http://www.umd.be/HSF) [17]. Sequence comparison data were obtained from Clustal Omega (http://www.ebi.ac.uk/Tools/msa/clustalo) [33]. Estimation of the open reading frame was done using NetStart 1.0 (http://www.cbs.dtu.dk/services/NetStart/). Functional analysis of the protein was performed using InterPro [18, 19] and ExPASy (http://web.expasy.org/compute_pi/).

### Statistical analysis

The ALLELE procedure of SAS/Genetics, version 9.4 (SAS Institute, Cary, NC, USA) was used to calculate polymorphism information content, heterozygosity, allelic diversity, allele and genotype frequencies and χ^2^-tests for Hardy-Weinberg-Equilibrium for the genotyped SNPs. The genome-wide association analyses were performed using a generalized linear model. The analyses were run using TASSEL [34]. The model explained for sex effects and the SNP genotypes. Further models with the first two to five principal components (PC) as covariates had been run to test the robustness of the models. PCs were parameterized to capture cryptic population structure. PCs were derived from the quality filtered set of SNPs. The results for the extended models were consistent with the model omitting PCs. We applied the Bonferroni correction using the MULTIPLE TEST procedure of SAS to determine the threshold for genome-wide significance. Quantile-quantile (Q-Q) plots for observed versus expected —log_10_P-values to be used for control of population stratification were calculated using SAS.

Distribution of genotypes was calculated using the FREQ procedure of SAS. Genotypic and allelic associations and allelic trends as well as odds ratios (ORs) with their 95% confidence intervals were calculated using the CASECONTROL procedure of SAS/Genetics. We analysed the haplotype structure of the identified genomic regions using Haploview 4.0 [35].

## Supporting Information

S1 Fig Pedigree of 15 ichthyosis-affected Great Danes. The affected dogs were derived from five different litters.
(TIF)

S2 Fig Histological findings in skin biopsies from Great Dane dogs. Skin sections from an unaffected neonatal puppy (A) and from a 28-days old puppy affected with ichthyosis (B). The skin of the affected puppy is characterized by marked epidermal and follicular hyperkeratosis (Hematoxylin and eosin staining, 100 x).
(TIF)

S3 Fig Linkage disequilibrium patterns (LD) for the SNP alleles in the region of 56–59 Mb. The LD coefficients (r2) between the markers are shown. Red fields display r² values greater than 0.50, white and blue fields display r² values less than 0.15. Two associated SNPs (BICF2S23340470, BICF2G630473300) can be found in a 134 kb haplotype block at 54 Mb and further four associated SNPs (BICF2G630473617, BICF2G630473695, BICF2G630473702, BICF2G630473744) are located in a haplotype block at 54,4–55 Mb of 343 kb length.
(TIF)

S1 Table Distribution of the six significantly associated SNPs in 22 Great Danes. All six SNPs (BICF2S23340470, BICF2G630473300, BICF2G630473617, BICF2G630473695, BICF2G630473702 and BICF2G630473744) show a perfect co-segregation with the phenotype.
(DOCX)

S2 Table Allele frequency in 420 samples of 35 different dog breeds and seven wolves. The number of dogs and wolves genotyped for the SNV *SLC27A4*:g.8684G>A of the *SLC27A4* gene and the frequency for the mutant A allele are shown. None of these control samples showed a genotype different from wild type.
(DOCX)

S3 Table Primer sequences used for validation of variants detected by sanger-sequencing. SNV *SLC27A4*:g.8684G>A was validated by the use of restriction fragment length polymorphism (RFLP) whereas the deletion *SLC27A4*:g.9852del could be investigated by gel-electrophoresis using the LI-COR automated sequencing system. Primer pairs, amplicon size (AS) in base pairs (bp), annealing (AT), restriction enzyme and incubation temperature (IT) are given.
(DOCX)

S4 Table Primer sequences for sequencing the genomic and cDNA of *SLC27A4*. Target regions, product sizes in base pairs (bp) and annealing temperatures (AT) are shown.
(DOCX)
