# Supplementary figures and images for: A Novel SLC27A4 Splice Acceptor Site Mutation in Great Danes with Ichthyosis
Source: PLoS One. 2015 Oct 27;10(10):e0141514. doi: 10.1371/journal.pone.0141514 (PMC4624637; doi:10.1371/journal.pone.0141514)

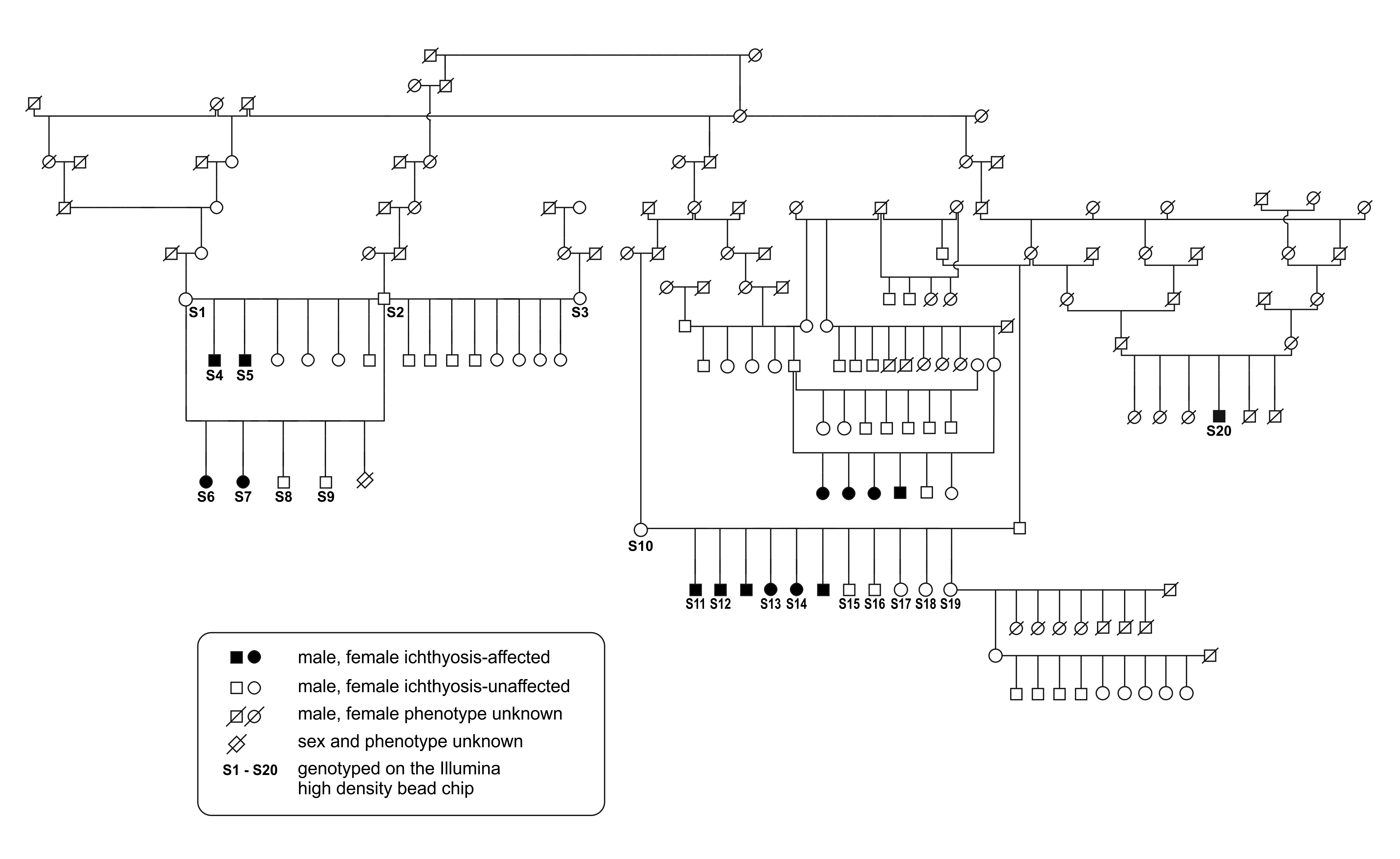

Supplement: S1 Fig — The affected dogs were derived from five different litters. (TIF) [file pone.0141514.s001.tif]

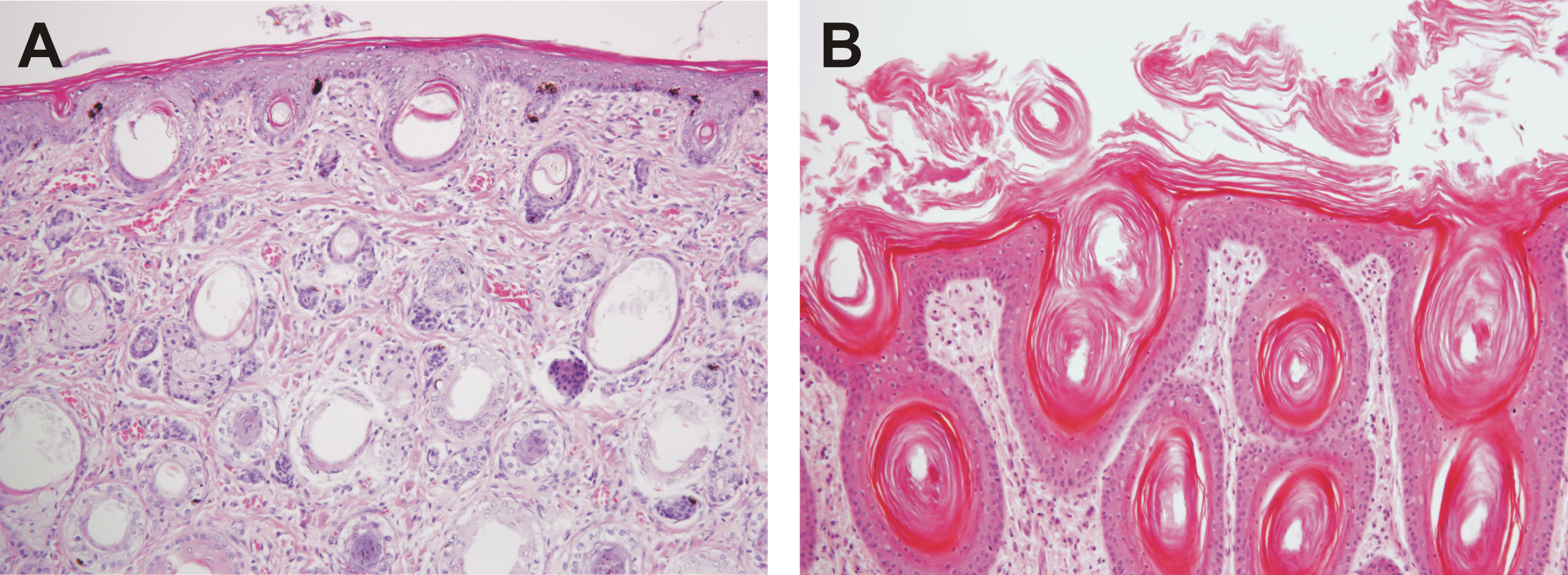

Supplement: S2 Fig — Skin sections from an unaffected neonatal puppy (A) and from a 28-days old puppy affected with ichthyosis (B). The skin of the affected puppy is characterized by marked epidermal and follicular hyperkeratosis (Hematoxylin and eosin staining, 100 x). (TIF) [file pone.0141514.s002.tif]

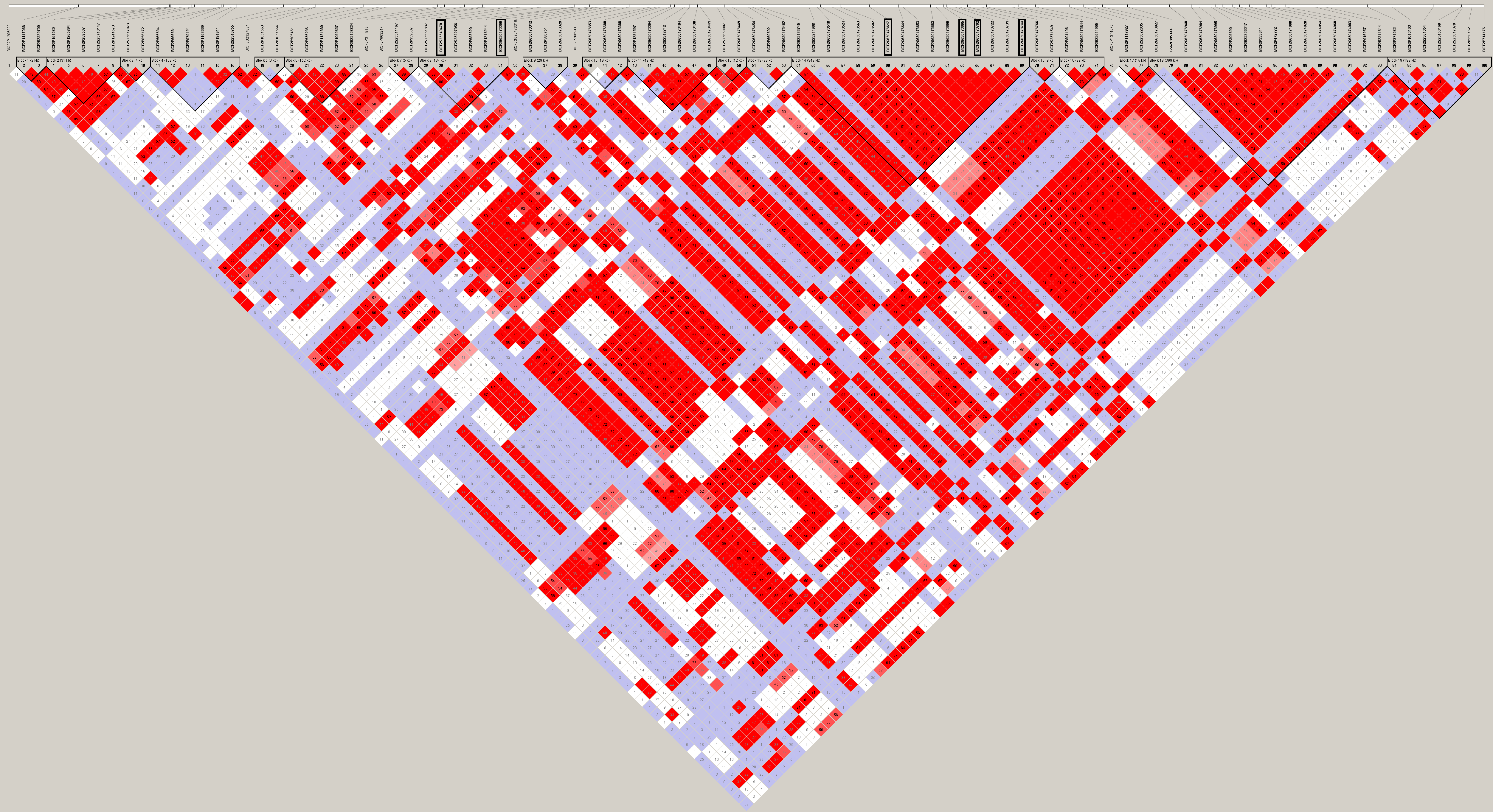

Supplement: S3 Fig — The LD coefficients (r2) between the markers are shown. Red fields display r² values greater than 0.50, white and blue fields display r² values less than 0.15. Two associated SNPs (BICF2S23340470, BICF2G630473300) can be found in a 134 kb haplotype block at 54 Mb and further four associated SNPs (BICF2G630473617, BICF2G630473695, BICF2G630473702, BICF2G630473744) are located in a haplotype block at 54,4–55 Mb of 343 kb length. (TIF) [file pone.0141514.s003.tif]
