# Supplementary material for: A Novel SLC27A4 Splice Acceptor Site Mutation in Great Danes with Ichthyosis
Source: PLoS One. 2015 Oct 27;10(10):e0141514. doi: 10.1371/journal.pone.0141514 (PMC4624637; doi:10.1371/journal.pone.0141514)
Supplement: S1 Table — All six SNPs (BICF2S23340470, BICF2G630473300, BICF2G630473617, BICF2G630473695, BICF2G630473702 and BICF2G630473744) show a perfect co-segregation with the phenotype. (DOCX) [file pone.0141514.s004.docx]

**S1 Table. Distribution of the six significantly associated SNPs in 22 Great Danes.** All six SNPs (BICF2S23340470, BICF2G630473300, BICF2G630473617, BICF2G630473695, BICF2G630473702 and BICF2G630473744) show a perfect co-segregation with the phenotype.

| Status | Genotype | | |
| --- | --- | --- | --- |
|  | 1/1 | 1/2 | 2/2 |
| Affected | 0 | 0 | 9 |
| Obligate carrier | 0 | 3 | 0 |
| Unaffected | 3 | 7 | 0 |
| Total | 3 | 10 | 9 |
