# Supplementary material for: A Novel SLC27A4 Splice Acceptor Site Mutation in Great Danes with Ichthyosis
Source: PLoS One. 2015 Oct 27;10(10):e0141514. doi: 10.1371/journal.pone.0141514 (PMC4624637; doi:10.1371/journal.pone.0141514)
Supplement: S2 Table — The number of dogs and wolves genotyped for the SNV SLC27A4:g.8684G>A of the SLC27A4 gene and the frequency for the mutant A allele are shown. None of these control samples showed a genotype different from wild type. (DOCX) [file pone.0141514.s005.docx]

**S2 Table. Allele frequency in 420 samples of 35 different dog breeds and seven wolves.** The number of dogs and wolves genotyped for the SNV *SLC27A4*:g.8684G>A of the *SLC27A4* gene and the frequency for the mutant A allele are shown. None of these control samples showed a genotype different from wild type.

| Breed | Number of samples | A Allele frequency |
| --- | --- | --- |
| Afghan Hound | 3 | 0 |
| Alaskan malamute | 1 | 0 |
| Appenzell Mountain Dog | 16 | 0 |
| Australian Shepherd | 8 | 0 |
| Bernese Mountain Dog | 40 | 0 |
| Boxer | 13 | 0 |
| Dalmatian | 18 | 0 |
| German Wirehaired Pointer | 15 | 0 |
| German Roughhaired Pointer | 5 | 0 |
| German Mittelspitz | 3 | 0 |
| German Pinscher | 3 | 0 |
| German Shepherd | 15 | 0 |
| Tibetan Mastiff | 13 | 0 |
| Doberman Pinscher | 16 | 0 |
| Dogo Argentino | 1 | 0 |
| Entlebuch Mountain Dog | 16 | 0 |
| Eurasier | 7 | 0 |
| European Wolf | 2 | 0 |
| Golden Retriever | 8 | 0 |
| Greater Swiss Mountain Dog | 15 | 0 |
| Havanese | 2 | 0 |
| Japanspitz | 2 | 0 |
| Kuvasz | 16 | 0 |
| Labrador Retriever | 8 | 0 |
| Norwegian Lundehund | 6 | 0 |
| Parson Russell Terrier | 10 | 0 |
| Patou | 2 | 0 |
| Wire-haired Dachshund | 15 | 0 |
| Saluki | 1 | 0 |
| Samojede | 10 | 0 |
| Shar-Pei | 95 | 0 |
| Shiba Inu | 1 | 0 |
| Siberian Husky | 9 | 0 |
| Tervueren | 2 | 0 |
| Tibetan Terrier | 16 | 0 |
| Timberwolf | 5 | 0 |
| Yorkshire Terrier | 2 | 0 |
| Total | 420 | 0 |
