# Supplementary material for: A Novel SLC27A4 Splice Acceptor Site Mutation in Great Danes with Ichthyosis
Source: PLoS One. 2015 Oct 27;10(10):e0141514. doi: 10.1371/journal.pone.0141514 (PMC4624637; doi:10.1371/journal.pone.0141514)
Supplement: S3 Table — SNV SLC27A4:g.8684G>A was validated by the use of restriction fragment length polymorphism (RFLP) whereas the deletion SLC27A4:g.9852del could be investigated by gel-electrophoresis using the LI-COR automated sequencing system. Primer pairs, amplicon size (AS) in base pairs (bp), annealing (AT), restriction enzyme and incubation temperature (IT) are given. (DOCX) [file pone.0141514.s006.docx]

**S3 Table. Primer sequences used for validation of variants detected by sanger-sequencing.** SNV *SLC27A4*:g.8684G>A was validated by the use of restriction fragment length polymorphism (RFLP) whereas the deletion *SLC27A4*:g.9852del could be investigated by gel-electrophoresis using the LI-COR automated sequencing system. Primer pairs, amplicon size (AS) in base pairs (bp), annealing (AT), restriction enzyme and incubation temperature (IT) are given.

| Gene | Polymorphism | Forward primer (5’-3’) | Reverse primer (5’-3’) | AS (bp) | AT (°C) | Restriction enzyme | IT  (°C) |
| --- | --- | --- | --- | --- | --- | --- | --- |
| *SLC27A4* | SLC27A4:g.8684G>A | TGTACGCCAAGACATTGCTG | TGACACTGGAAAAGCTCTGG | 670 | 60 | MspI | 37 |
| *SLC27A4* | SLC27A4:g.9852Del | CAGGGTGGAGAGAAATGAAGG | GTACAGGTAGCCCAGCTCATC | 186 | 60 | - | - |

AS: amplicon size; AT: annealing temperature; IT: incubation temperature
