# Supplementary material for: A Novel SLC27A4 Splice Acceptor Site Mutation in Great Danes with Ichthyosis
Source: PLoS One. 2015 Oct 27;10(10):e0141514. doi: 10.1371/journal.pone.0141514 (PMC4624637; doi:10.1371/journal.pone.0141514)
Supplement: S4 Table — Target regions, product sizes in base pairs (bp) and annealing temperatures (AT) are shown. (DOCX) [file pone.0141514.s007.docx]

**S4 Table. Primer sequences for sequencing the genomic and cDNA of *SLC27A4.*** Target regions, product sizes in base pairs (bp) and annealing temperatures (AT) are shown.

| Gene | Gene region | Forward primer (5’-3’) | Reverse primer (5’-3’) | Product size (bp) | AT (°C) | genomic or coding DNA-Primer |
| --- | --- | --- | --- | --- | --- | --- |
| *SLC27A4* | exon1 | TTGGATGAGGAGTCAAACCAG | CCTAAGCTCAACAGCACCAAG | 826 | 60 | genomic |
| *SLC27A4* | exon2 | CAGTCACAGCTCCCTTTCTTG | AAGAATCGCTACCACTGCATC | 720 | 60 | genomic |
| *SLC27A4* | exon3 | GTGAGAGAGCCAGCTTGGAG | ATGATGGATGGCAATGAAGG | 741 | 60 | genomic |
| *SLC27A4* | exon4-5 | CCCTGTGCTGTCTTTTCTCAC | CATCACTGGGCTCTTCTCTTG | 772 | 60 | genomic |
| *SLC27A4* | exon6 | GGTGCTTAGAGTCAGGTGTGG | AATGTTGCTCCCAAATGAGTG | 725 | 60 | genomic |
| *SLC27A4* | exon7-8 | TGTTCTGATGGGGAGACTGAG | AGGGTCCTTCACACAACAGTG | 808 | 60 | genomic |
| *SLC27A4* | exon9-11 | TCTGTGGGAAGCACTGTGAC | CTGGAGCCCTGGAAACATAC | 850 | 60 | genomic |
| *SLC27A4* | exon12-13 | ATGATGCCAGTGACCAAGAC | GGCCCTTAGAGGGAGTCAGG | 850 | 60 | genomic |
| *SLC27A4* | 5’UTR-exon 2 | GAGAACCTCCTTGGGTCTTTG | GCCTTGACCTTCAGGAGTACC | 667 | 60 | coding |
| *SLC27A4* | exon 1-4 | GTTTCTCTACCTGGGGTCTGG | CCTGTGGTACCCGATGTGTAG | 663 | 60 | coding |
| *SLC27A4* | exon 2-6 | TTGATCTTTGAGGGCACAGAC | TGATACAGTCGTCCCAGAACC | 697 | 60 | coding |
| *SLC27A4* | exon 7-11 | TTCTAGCCGTTTCCACATCC | GTGCAGCTCAGGAAGGAAGC | 825 | 60 | coding |
| *SLC27A4* | exon 11-3’UTR | GTTTTAACCCGGCAGTTGTG | GAGGTGCAGGCAATCTTGAG | 603 | 60 | coding |
| *SLC27A4* | exon8-10 | GCATCCTGTCCTTTGTGTACC | GTGCAGCTCAGGAAGGAAGC | 545 | 61.5 | coding |

AT: Annealing temperature
